# Arachidonic acid mediates the formation of abundant alpha-helical multimers of alpha-synuclein

**DOI:** 10.1038/srep33928

**Published:** 2016-09-27

**Authors:** Marija Iljina, Laura Tosatto, Minee L. Choi, Jason C. Sang, Yu Ye, Craig D. Hughes, Clare E. Bryant, Sonia Gandhi, David Klenerman

**Affiliations:** 1 Department of Chemistry, University of Cambridge, Lensfield Road, Cambridge CB2 1EW, UK; 2 Department of Molecular Neuroscience, University College London, Institute of Neurology, Queen Square, London WC1N 3BG, UK; 3 Department of Cell Biology, Harvard Medical School, Boston, 02115, USA; 4 Department of Veterinary Medicine, University Of Cambridge, Madingley Road, Cambridge, CB3 0ES, United Kingdom.

## Abstract

The protein alpha-synuclein (αS) self-assembles into toxic beta-sheet aggregates in Parkinson’s disease, while it is proposed that αS forms soluble alpha-helical multimers in healthy neurons. Here, we have made αS multimers *in vitro* using arachidonic acid (ARA), one of the most abundant fatty acids in the brain, and characterized them by a combination of bulk experiments and single-molecule Fӧrster resonance energy transfer (sm-FRET) measurements. The data suggest that ARA-induced oligomers are alpha-helical, resistant to fibril formation, more prone to disaggregation, enzymatic digestion and degradation by the 26S proteasome, and lead to lower neuronal damage and reduced activation of microglia compared to the oligomers formed in the absence of ARA. These multimers can be formed at physiologically-relevant concentrations, and pathological mutants of αS form less multimers than wild-type αS. Our work provides strong biophysical evidence for the formation of alpha-helical multimers of αS in the presence of a biologically relevant fatty acid, which may have a protective role with respect to the generation of beta-sheet toxic structures during αS fibrillation.

The aggregation of alpha-synuclein protein (αS) into amyloid fibrils is implicated in Parkinson’s disease (PD), Dementia with Lewy Bodies (DLB) and other synucleopathies^1^. Fibrils of αS are the major constituents of insoluble deposits, Lewy bodies and Lewy neurites, found in the brains of PD and DLB patients^2^. Moreover, small oligomers of αS formed early during the process of fibril formation are believed to represent the most toxic forms of this protein that cause irreversible neuronal damage^3^,^4^,^5^.

Apart from being disease-related, αS is also highly abundant in healthy brain tissue and constitutes around 1% of the total brain proteins^6^. While in the pathogenic aggregates αS acquires beta-sheet conformation, its normal physiological state remains a subject of debate and active research. It has been demonstrated to be either purely monomeric in human tissue^7^ and neuronal cells^8^, or to form alpha-helically folded tetramers and related multimers^9^,^10^,^11^,^12^. In the brain, αS is localized to presynaptic nerve terminals in close proximity to synaptic vesicles^13^, and is involved in the transmission of vesicular cargo^14^, as well as vesicle trafficking and retrieval^15^. These processes occur via phospholipid membrane-based mechanisms^16^,^17^,^18^,^19^, implying a connection between the normal function of αS in healthy neurons with phospholipids and fatty acids (FAs). Furthermore, αS shares structural homology with the family of fatty acid binding proteins and is able to bind FAs^20^. Arachidonic acid (ARA) is a polyunsaturated FA and is one of the most abundant FAs in gray matter phospholipids in the human brain^21^. ARA is continuously released from the phospholipid membranes into the cytosol of brain neurons by the action of enzymes^22^, where it may co-exist with αS. Several *in vitro* studies have observed that ARA was able to promote the self-assembly of αS into a range of aggregates under various incubation conditions^23^,^24^,^25^,^26^.

Alpha-helically-folded multimers of αS, identified in living cells, have been difficult to prepare *in vitro* due to their dynamic nature^27^ and scarce information on other potentially required stabilizing factors, such as membrane binding^28^. A rapid change of αS from unstructured to alpha-helical conformation was observed in the presence of ARA^26^ and related fatty acids^29^, upon interaction with lipid membranes^30^, detergent micelles^31^ and lipid vesicles^32^,^33^,^34^. However, to date, it has not been possible to characterize the formation of small alpha-helically folded multimers at physiologically-relevant concentrations, owing to the low abundance of these species and the difficulties in contrasting them to the disease-related oligomers. To address this, we prepared alpha-helically-folded multimers of αS in the presence of ARA, and compared them to the toxic oligomers of αS using single-molecule Fӧrster resonance energy transfer (sm-FRET) measurements. We present a set of comparative assays to investigate the differences in the stabilities of both types of aggregates.

## Results

### Single-molecule FRET experiments

The main technique employed in this work is single-molecule FRET (sm-FRET). This highly sensitive method is particularly suitable for the characterization of the oligomers of αS, typically present in solutions in low abundance and in a vast excess of monomeric protein. In our previous related studies, this method was utilized to characterize the formation of oligomers during the aggregation of αS in aqueous solution in unprecedented detail^35^,^36^,^37^,^38^. Moreover, the sm-FRET method was further improved by performing the measurements under steady flow, which allowed to observe the formation of oligomers of αS with higher time resolution^36^. This latter improvement enabled to study a rapid self-assembly of αS in the presence of ARA in this study.

For sm-FRET experiments, full-length αS protein was used, and an alanine to cysteine mutation was introduced at residue 90 (A90C). This allowed the attachment of a single fluorophore, Alexa Fluor (AF) dye, per protein molecule. The mutation was demonstrated not to significantly affect the aggregation properties of αS in our previous studies^35^,^36^,^37^,^38^, because the residue 90 is located at the periphery of the beta-sheet core. For all sm-FRET aggregation assays, 1:1 stoichiometric ratio of αS labeled with Alexa Fluor 488 (αS-AF488) and Alexa Fluor 594 (αS-AF594) was used, and ARA was unlabeled. The solutions were incubated, and aliquots were withdrawn and immediately diluted to picomolar concentrations of the protein, allowing the analysis in single-molecule regime, before being flowed through a microfluidic channel and irradiated with a focused 488 nm laser beam, as described previously^36^. Subsequently, the fluorescence signal was recorded separately in the AF488 (donor) and the AF594 (acceptor) channels. The 488 nm beam directly excites αS-AF488 molecules. Thus, αS-AF488 monomers are observed in the experiment as single fluorescence bursts in the donor channel, whereas αS-AF594 monomers remain undetected. Since oligomers contain both αS-AF488 and αS-AF594 molecules, the directly excited αS-AF488 non-radiatively excites the αS-AF594 via FRET process. Oligomers are therefore observed as simultaneous fluorescence intensity bursts in both the donor and the acceptor channels (Supplementary Fig. 1), and thus are distinguished from the excess of the monomeric protein in solution, and the numbers of detected oligomers are determined. In this method, the fluorescence intensity values of the oligomeric bursts were used to derive a FRET efficiency value for each oligomer (equation 1 in Methods), and its apparent size (equation 2 in Methods). The FRET efficiency values of oligomers are related to their compactness, while the determination of the apparent sizes allows monitoring the growth of aggregates over time, as well as excluding larger fibrillar species, as described in detail in Methods.

### The presence of ARA results in a rapid self-assembly of αS in solution

**Figure 1:**
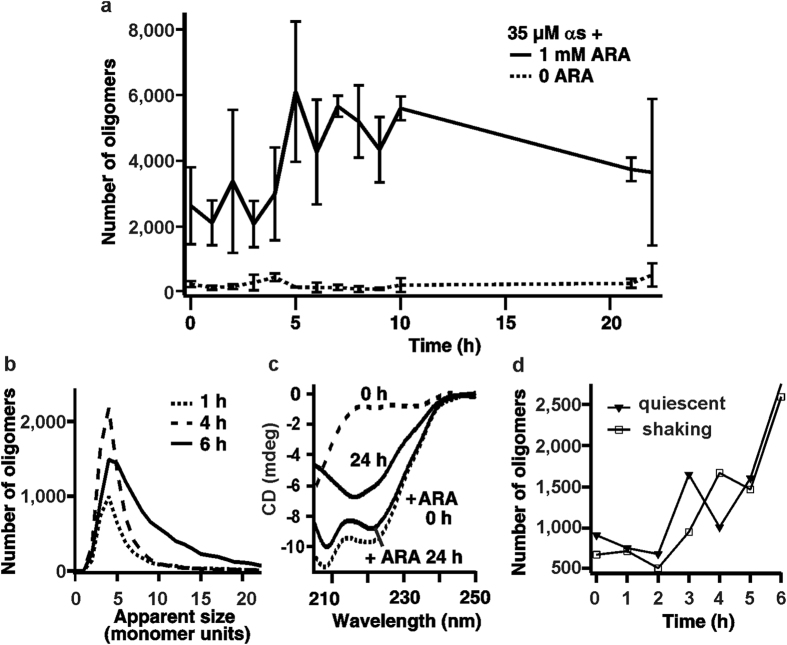
Effect of ARA on αS. (**a)** Kinetic profile of αS aggregate formation induced by ARA at quiescent conditions, and of αS aggregation in pure buffer under quiescent conditions (n = 3, std). (**b)** Time evolution of the apparent size histograms indicating growth of the aggregates. (**c)** CD spectra of αS-AF594 with and without ARA. Without the acid, at 0 h protein is mostly in random-coil conformation, and contains beta-sheet structure after 24 h with shaking, indicated by a broad negative band at 217 nm. In the presence of ARA, both shortly after addition and after 24 h of incubation, CD spectra show the features corresponding to the alpha-helical state, negative bands at 208 nm and 221 nm. (**d)** Kinetic profile of aggregate formation of 35 μM αS in the presence of 1 mM ARA under either shaking or non-shaking conditions (n = 3). Comparable results suggest the absence of the effect of shaking on the kinetics in the presence of ARA.

The self-assembly of αS was monitored in solutions of 35 μM αS in the presence of 1 mM concentration of ARA, above the critical micellar concentration (CMC) of the acid^26^,^39^. The samples were incubated under quiescent conditions, either in the presence or in the absence of ARA. Since the aggregation of pure αS in physiological buffer is known to be inefficient at quiescent conditions *in vitro*^40^, the oligomerization at 35 μM concentration of αS in the absence of ARA was negligible, and only a small increase in the numbers of detected oligomers was observed over a 24-hour incubation (Fig. 1a). In contrast, 35 μM αS incubated with 1 mM ARA showed a rapid formation of oligomers, judged by the presence of high levels of detected FRET events, recorded shortly after the addition of ARA (Fig. 1a). We confirmed the absence of any background fluorescence from the ARA solution in buffer. In addition, there was no apparent effect of ARA on the fluorescence signal from the fluorophores in the sm-FRET experiments, which was confirmed by performing control measurements using dual-labeled (AF488 and AF594) 40-base pair DNA samples, as detailed in Supplementary Fig. 2. Therefore, the initial rise in coincident events upon the addition of ARA to αS solution was due to the self-assembly of αS into multimers, which we will further refer to as ‘ARA-induced oligomers’. The increase in the numbers of these species continued most rapidly for 6 hours, followed by a plateau and a subsequent slight decrease after 20 hours (Fig. 1a), which could be due to a partial disaggregation of the species or, alternatively, due to their higher-order association. In addition to the rise in the numbers of multimers with time, there was an identifiable increase in their average apparent sizes, observed from the broadening of the apparent size distributions (Fig. 1b). Typically, there was a progression towards larger species within first 6–7 hours of the experiment, and no subsequent change, suggesting that their formation reached a steady state within this time period.

Circular dichroism (CD) measurements showed that the fluorescently labeled αS was intrinsically disordered in the absence of ARA at the beginning of the experiment, and acquired beta-sheet conformation after 24 hours of incubation, particularly in the samples incubated under shaking conditions, which overall indicated that the labeled protein could assemble into fibrils as in our previous works^35^,^38^. In contrast, the spectra of the samples in the presence of ARA were characteristic of alpha-helical conformation shortly upon addition of ARA, and after 24 hours of incubation. The observed attainment of alpha-helical conformation by αS in the presence of ARA is in agreement with previously reported results for αS with ARA^26^, as well as for αS in the presence of the excess of a structurally similar docosahexaenoic acid^29^. Generally, the transition from the disordered to alpha-helical state is typical for αS upon its association with lipid membranes^16^,^18^, and suggests that αS binds ARA at our experimental conditions. To check whether the formation of αS oligomers, promoted by ARA, occurred when shear forces were introduced, we performed incubations with shaking as well as under quiescent conditions, which yielded comparable results at the same starting concentrations of αS and ARA (Fig. 1d). The quiescent preparation using 35 μM αS and 1 mM ARA was found to be the most high-yielding among other tested preparations, including incubations using different isoforms of αS or varying concentrations of both αS and ARA, as detailed in Supplementary Methods and Supplementary Figs 3 and 4.

### Further evidence for the differences between ARA-induced oligomers and oligomers formed in its absence

Once the ARA-induced oligomers of αS were observed, we sought to compare these multimers to the toxic oligomers of αS which are formed by the protein in aqueous buffer. Using sm-FRET, we previously demonstrated that αS assembles into toxic beta-sheet-rich oligomers in aqueous buffer under constant agitation^35^,^36^,^38^. Therefore, here we used the same strategy as before to prepare these αS-only oligomers (see Supplementary Information for details), and carried out a series of comparative experiments using ARA-induced oligomers and αS-only oligomers.

**Figure 2:**
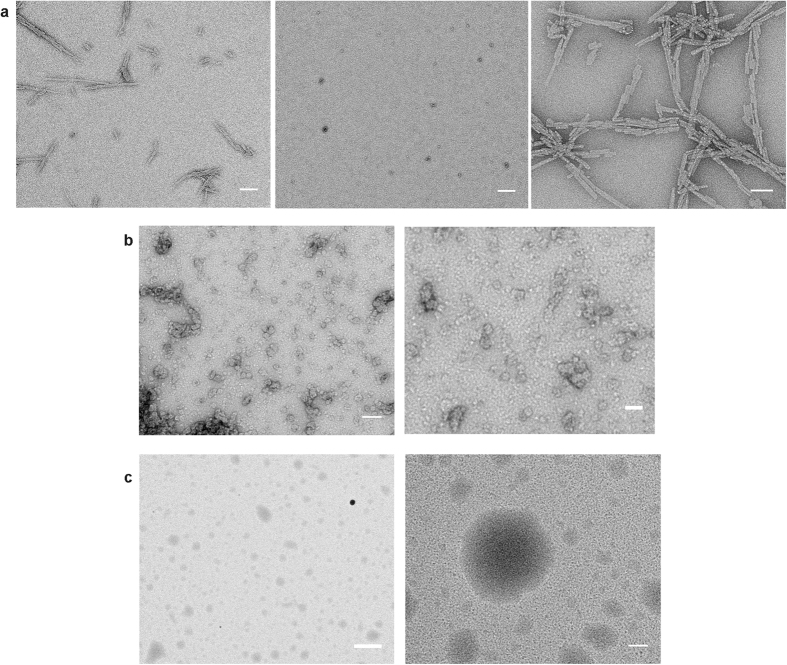
TEM images of aggregates. (**a**) αS aggregates in buffer (35 μM, 24 h, shaking). From left to right: before centrifugation (15 min at 14.2 r.p.m.), a mixture of oligomers and fibrils is observed; soluble oligomers are present in the supernatant after centrifugation; insoluble pellet after centrifugation contains fibrillar aggregates. Scale bars (left to right) 200 nm, 200 nm and 100 nm. (**b)** ARA-induced oligomers of αS (35 μM, 1 mM ARA, 24 h, non-shaking). No fibrils observed after centrifugation. Abundant soluble oligomers and oligomer agglomerates are present. Scale bars 100 nm (left) and 50 nm (right). (**c)** ARA acid micelles in buffer (1 mM ARA) in the absence of αS. Scale bars 1 μm (left) and 100 nm (right).

Firstly, we found using transmission electron microscopy (TEM) that the ARA-induced oligomers had markedly different morphologies compared to the oligomers of αS formed in aqueous buffer (Fig. 2). In the case of αS samples in buffer solution, after 24 hours of incubation the solutions contained a mixture of monomers, soluble oligomeric species as well as insoluble fibrils (Fig. 2a). The majority of the insoluble fibrils could be removed by centrifugation, as judged by TEM and confirmed in our previous work^38^, therefore this step was introduced for the preparation of the αS-only oligomers. In the case of the samples with ARA-induced oligomers, abundant populations of oligomers were observed, frequently associated into higher-order assemblies (Fig. 2b). Interestingly, these aggregates were soluble, judged from the absence of fibrillar aggregates in TEM images and no precipitate upon centrifugation of the samples. In the absence of αS, at 1 mM ARA, micelles were formed (Fig. 2c). In the presence of the protein, however, the aggregates visualized using TEM were smaller compared to the micellar structures, which is consistent with the previously reported observation that αS is able to disrupt the micelles of ARA^26^. Overall, ARA-induced oligomers were larger and had less regular shapes than the αS-only oligomers, probably due to the association with the FA, and had a tendency to assemble into higher-order soluble agglomerates. They looked similar in morphology to the previously reported oligomers of αS in the excess of docosahexaenoic acid^29^.

**Figure 3:**
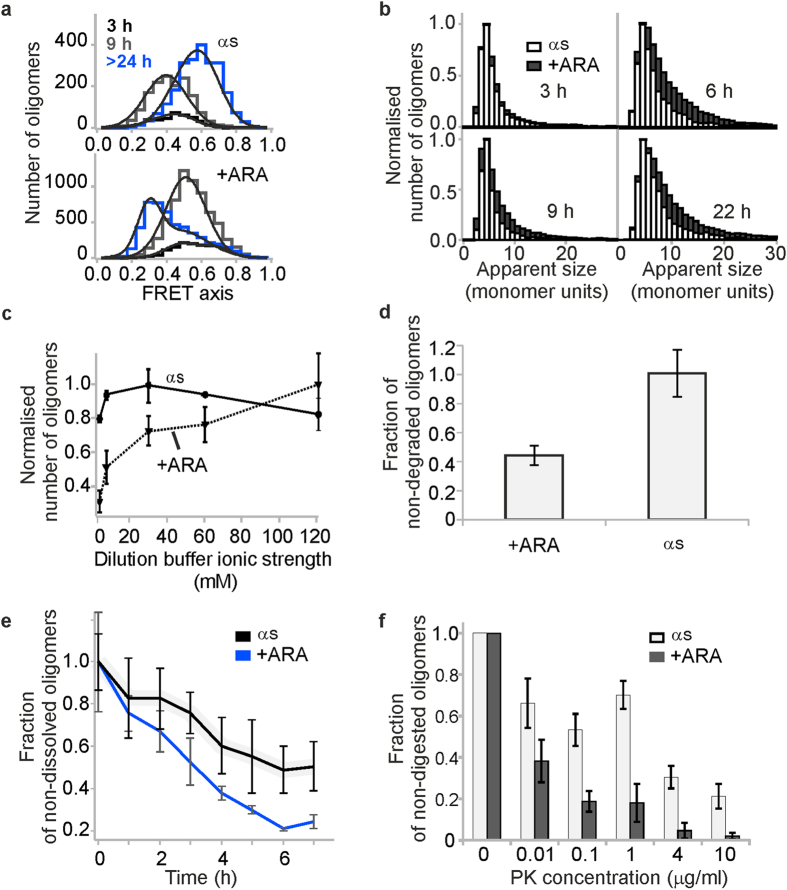
Comparative sm-FRET experiments to investigate the differences between ARA-induced oligomers and oligomers formed in aqueous buffer during 24 h of incubation. (**a)** Appearance and time evolution of FRET efficiency histograms. Fits to eq. 3, with FRET eficiency values (E) of E(αS 9h) = 0.4±0.01, E(αS 24h) = 0.57±0.01, E(αS ARA 9h) = 0.52±0.01, E(αS ARA 24h) = 0.301±0.003 and 0.48±0.03. (**b)** Comparison of apparent size distributions. (**c)** Salt gradient measurements (n = 3, std). (**d)** Degradation by 26S proteasome over 12 h (n=3, std). (**e)** Oligomer disaggregation upon dilution into aqueous buffer to 280 pM (n = 3, std). (**f)** Comparative dose-response assay of Proteinase K digestion (n = 3, std).

Having determined by TEM that ARA-induced oligomers had different morphologies compared to αS-only oligomers, we further investigated the differences between these types of species (Fig. 3). Firstly, a closer inspection of the FRET efficiency histograms revealed clear differences (Fig. 3a). As it has already been mentioned, using sm-FRET method we previously characterized the oligomerization process of αS in buffer solution in great detail^35^,^36^,^37^,^38^. In these previous studies, we reproducibly observed the conversion of initially formed disordered oligomers of αS to a more stable and compact form, associated with the highest cytotoxicity. These two types of oligomers could be distinguished based on their FRET efficiency values (eq. 1). While the initially formed disordered oligomers had low FRET efficiency values, and thus termed ‘low-FRET’, the stable toxic oligomers had characteristic high FRET efficiency values, and were termed ‘high-FRET’ oligomers. Here, the FRET efficiency histograms obtained for the samples of αS-only oligomers were in good agreement with the previous results. As in previous studies, after 9 hours of incubation mainly disordered oligomers were present with the mean FRET efficiency value E = 0.4, whereas after 24 hours the majority of the population contained the stable oligomers with high FRET values, E = 0.6. In contrast, ARA-induced oligomers gave rise to FRET efficiency histograms that showed a single peak with E value of 0.5 after 9 hours of incubation, and a broad FRET distribution after 24 hours. Since the appearance of FRET efficiency histograms was found to be well-correlated with the stability of oligomeric aggregates in our previous work^35^, the observed differences of the FRET efficiency histograms of the ARA-induced multimers compared to αS oligomers in buffer solution could suggest differences in their stabilities, and possibly differences in their structures. In addition, αS oligomers formed in the presence of ARA were found to be larger in terms of average numbers of monomers per oligomer, particularly at later incubations times past 6 hours of incubation, as shown in Fig. 3b.

To explore whether these differences in the FRET histograms and apparent size distributions of the ARA-induced oligomers compared to αS-only oligomers were associated with the differences in oligomer stabilities, we carried out more comparative experiments. In our previous work, it was found that the high-FRET oligomers were more stable with respect to dilution into low ionic strength buffer in comparison to their preceding disordered oligomers. We therefore compared the stability of ARA-induced multimers and αS-only (high-FRET) oligomers to the changes in ionic strength. Samples were diluted into buffers with varying ionic strength and the numbers of oligomers in solution were counted using sm-FRET. The result showed that the αS-only high-FRET oligomers prepared in buffer were stable with respect to the changes in ionic strength, which agreed with our previous results. In contrast, ARA-induced oligomers dissociated to a greater extent at low ionic strengths, which is probably due to the lack of a beta-sheet structure in these species.

Next we investigated whether the lower structural stability of ARA-induced αS oligomers could facilitate their enzymatic degradation. The 26S proteasome is the main protein degradation machinery in eukaryotic cells, including degradation of αS monomers upon modification by ubiquitin^41^, yet it was reported to be inactive to certain αS aggregates^42^. We have recently observed a rapid *in vitro* degradation of αS monomers independently of ubiquitin modification, and found that αS oligomers with a specific type of ubiquitin modification alter the structural properties to enable direct proteasomal degradation (Y. Y.). In order to compare the susceptibilities of the ARA-induced oligomers and the αS-only oligomers towards degradation by the proteasome, comparative assays were carried out, using identical degradation mixtures and incubation conditions for both kinds of oligomers and exposing both types of aggregates to mammalian 26S proteasome for a period of 12 hours (Fig. 3d). Over this selected incubation time, the numbers of ARA-induced oligomers were reduced upon proteasome-treatment. In contrast, the samples lacking ARA were not targeted by the proteasome, in agreement with the previous literature. This suggested that ARA-induced oligomers were more degradable by proteasome in comparison to the αS-only oligomers.

To confirm that ARA-induced oligomers were less stable than αS-only oligomers, we carried out oligomer disaggregation experiments, where the oligomeric samples were diluted to single-molecule concentrations into buffer, and left under quiescent conditions for several hours. During this time, the decrease in the numbers of oligomers due to their dissociation into monomers in solution was monitored by sm-FRET (Fig. 3e). While the decrease in the numbers of oligomers was observed for both the ARA-induced oligomers and the oligomers formed in its absence, it was faster for the former species confirming the previous findings of their lower stability. This difference points towards structural differences of the ARA-induced oligomers compared to αS-only oligomers.

To gain further information about this difference, comparative proteolytic digestion assays were carried out, by exposing both the ARA-induced oligomers and the oligomers formed in the absence of ARA to varying concentrations of Proteinase K (PK) enzyme (Fig. 3e). Our previous studies showed that high resistance to PK digestion was characteristic for beta-sheet conformation in αS aggregates, and amyloid fibrils were the most resistant in this assay^35^. Here, both types of oligomers were susceptible to PK digestion; however, the fractions of remaining non-digested oligomers were consistently higher in the samples containing αS-only oligomers, additionally indicating that the ARA-induced oligomers were less stable, consistent with the lack of beta-sheet structure in these aggregates.

**Figure 4:**
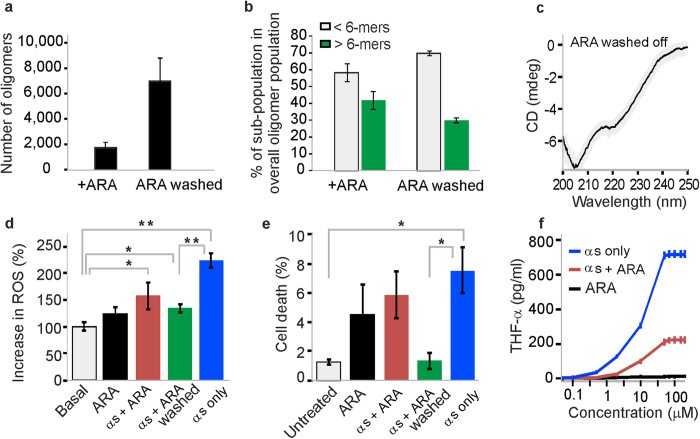
ARA depletion experiments and comparative cell assays. (**a)** Numbers of oligomers, detected by sm-FRET before and after decreasing the concentration of ARA by washing with excess buffer (see Methods) (n = 3, std). (**b)** The increase in the smallest oligomers (2-5-mers) is observed, whereas the fraction of larger species drops, indicating that oligomers undergo a partial dissociation upon separation from the acid. **(c)** CD spectrum acquired after washing the protein sample, showing that the alpha-helical conformation is preserved. The detection of intact small multimers and alpha-helical conformation indicate that ARA is still present in solution and bound to αS in these multimers. Thus, it is very difficult to fully separate the FA from αS under these conditions. (**d)** Cytoplasmic ROS production by monitoring the rate of the ratio of the oxidised to reduced form of dihydroethidium (n = 50–90 cells, sem). Application of αS oligomers (500 nM of total αS) lead to a significant increase in ROS production (222 ± 12.95% compared to 100% basal, n =  88 cells, P < 0.01 relative to basal level). Application of ARA-induced oligomers (500 nM of total αS) showed small increase in ROS generation (134 ± 7.78%, n = 73 cells, P < 0.05 relative to basal level). Application of washed ARA-induced oligomers after ARA depletion by centrifugation (500 nM of total αS) or application of ARA alone (14.2 μM ARA) produced close to basal levels of ROS. (**e)** Percentage of cell-death as measured by Hoechst/propidium iodide staining after overnight incubation with the αS-only or ARA-induced oligomers, or ARA (n = 6–9 fields of view, sem). αS-only oligomers caused an increased cell death (7.54 ± 1.57%, n(cells)  =  693, P < 0.05 relative to untreated group), while ARA-induced oligomers washed from excess ARA lead to basal levels of cell death (1.3 ± 0.52%, n  =  7 fields of view, n(cells) = 509). (**f)** Pro-inflammatory response measured by the production of THF-α in BV2 microglia after a 24-h incubation of the cells after treatment with αS-only oligomers, ARA-induced oligomers and ARA alone, added at a range of concentrations between 0.05–200 μM (n = 4, sem).

Lastly, we investigated whether ARA itself was a constituent of the ARA-induced oligomers. Due to the observed tendency to assemble into large agglomerates (Fig. 2b), it was presumed that the association of ARA-induced oligomers into larger aggregates could occur via the free fatty acid molecules, and we set out to test whether these species could remain stable upon decreasing the concentration of ARA in solution. To address this, ARA- and αS-containing samples were prepared as described above, and the concentration of the acid was decreased by washing with the excess of aqueous buffer and by subsequently concentrating the protein solutions, as described in Methods. This resulted in the increase in the numbers of recovered oligomers (Fig. 4a), as well as an 11 ± 5% increase in the population of small species consisting of less than 6 apparent monomer units, and a drop in the sub-population of larger oligomers (Fig. 4b). This indicated that the multimers had undergone a partial dissociation during the process (Fig. 4b), suggesting that the excess of fatty acid molecules acts to stabilize the larger multimers. Nevertheless, the finding that the majority of the aggregates could be recovered and remained sufficiently stable to be detected at picomolar concentrations of the protein in the sm-FRET experiments implies a degree of stability, and indicates a strong binding of ARA to αS in these aggregates. These observations are compatible with the previous reports of the stability of the FA-induced multimers of αS upon chromatographic procedures^23^,^29^, and highlight a challenge of removing FAs from αS under these conditions. The result that the oligomers partially dissociated upon the decrease of FA suggests that ARA is a stabilizing constituent of these aggregates, which is consistent with previous related findings that αS co-aggregated with anionic lipids^43^. Consistent with this, the CD spectrum of αS solution, recorded after decreasing the concentration of ARA, indicated that the alpha-helical conformation was preserved in the samples (Fig. 4c).

### ARA-induced oligomers of αS are less damaging to cells than oligomers formed in its absence

In order to further compare ARA-induced oligomers with the oligomers formed in its absence, we investigated their relative abilities to cause cell damage. We have previously reported that αS oligomers that are generated in aqueous buffer under constant shaking can promote the production of reactive oxygen species (ROS) when applied to primary neuronal cultures^35^. To assess the extent of ROS production promoted by ARA-induced oligomers, we measured the production of superoxide induced by their application to primary cultures of cortical neurons by quantifying the rate of oxidation of the dye dihydroethidium, as detailed in Methods and Supplementary Information, and shown in Fig. 4d. In these experiments, αS oligomers (500 nM total αS) prepared in the absence of ARA lead to the highest significant increase in the production of ROS relative to the basal level, 222 ± 12.95% compared to 100% basal (P < 0.01), in agreement with our previous reports^35^,^38^. In contrast, application of the same concentration of ARA-induced oligomers after the depletion of excess free ARA produced a smaller increase in ROS, 134 ± 7.78% in comparison to the 100% basal level (P < 0.05), which was significantly reduced in comparison to what was shown by oligomers prepared in the absence of ARA (P < 0.01). As controls, we utilized ARA alone and ARA-induced oligomers without depletion of ARA, and in both cases again observed only a small increase in ROS production over basal levels (P < 0.05) (Fig. 4d).

In addition, we investigated the ability of ARA-induced αS oligomers to promote cell death, as described in Methods and Supplementary Information. Representative images from these experiments are in Supplementary Fig. 5. We found that the incubation of cells overnight with αS oligomers generated in aqueous buffer induced a significant increase in cell death (P < 0.05), as detailed in Fig. 4e, corroborating their previously established neurotoxic nature^35^. Conversely, incubation of cells with ARA-induced oligomers did not induce a significant increase in basal levels of cell death, shown in Fig. 4e. We observed that free ARA was able to cause cell damage in both of these assays, particularly upon longer incubations presumably due to its oxidation, while ARA-induced oligomer samples after depletion of the excess of ARA were benign in the cell death assay. Overall, these results suggest that ARA-induced oligomers are less toxic to the cortical neurons than αS oligomers generated in its absence, particularly under the conditions when excess free ARA is rapidly depleted. This result may have physiological relevance, given that free ARA is known to be highly transient *in vivo*^44^.

Furthermore, we tested whether ARA-induced oligomers could lead to a reduced pro-inflammatory activation of microglia relative to the αS oligomers prepared in its absence. Growing evidence suggests that the activation of microglia is linked to the progression of neurodegeneration in synucleophathies^45^. We assessed the response of microglia by measuring the concentration of secreted tumor necrosis factor alpha (TNF-α) protein, which is one of the major pro-inflammatory cytokines that is released by microglia upon their activation, and has been shown to promote αS-induced cell death^46^. In these experiments, detailed in the Methods section, the secretion of TNF-α was found to be consistently higher in response to the oligomers formed in aqueous buffer than in response to the ARA-induced oligomers, and this difference was clearly observed at a broad range of applied total concentrations of αS (Fig. 4f).

### ARA-induced oligomer formation at physiologically-relevant concentrations of αS and ARA

**Figure 5:**
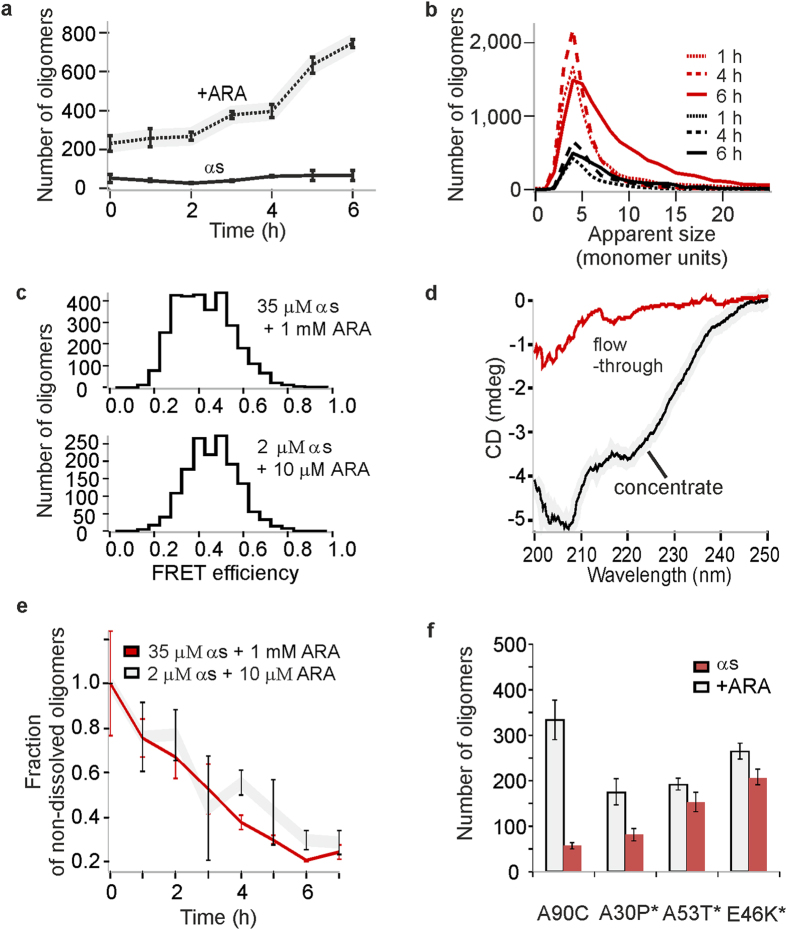
Experiments using physiological concentrations of αS and ARA. (**a)** Kinetic profile of oligomer formation at 2 μM αS in the presence of 10 μM ARA (dashed line), and in the absence of ARA under the same conditions (black line) (n = 3, std). (**b)** Comparison of apparent size distributions at 35 μM αS with 1 mM ARA (red), or 2 μM αS with 10 μM ARA (black). (**c)** Representative FRET efficiency histograms, resulting from sm-FRET analysis of the oligomers formed with either 35 μM αS with 1 mM ARA, or 2 μM αS with 10 μM ARA after 6 h. After this and later times, there was no difference in the appearance of the histograms, apart from lower total numbers detected in the lower-concentration samples, when the same protein concentration was used for the detection. (**d)** CD spectra of the solutions of 2 μM αS with 10 μM ARA after 24 h and enrichment using 100 kDa spinfilter. (**e)** Overlaid oligomer disaggregation profiles upon dilution into aqueous buffer to 280 pM of 35 μM αS samples with 1 mM ARA (red), and 2 μM αS with 10 μM ARA (grey) (n = 3, std). (**f)** Numbers of oligomers detected after >30 h using a range of αS isoforms, either A90C or pathological mutants, at 2 μM with 10 μM ARA (grey) or in the absence of ARA (red) (n = 6, sem).

In the above experiments ARA has been used at high concentration above its CMC value^26^,^39^. Because ARA is a biologically relevant molecule and occurs *in vivo* at concentrations significantly below its CMC, we extended our study to more physiologically-relevant concentrations of 2–10 μM^47^ (Supplementary Fig. 6a). In addition, the concentrations of αS protein at the synapse were reported to be in the range 2–5 μM^11^. Therefore, in order to mimic the concentrations of both αS and ARA found *in vivo*, we combined 2 μM αS and 10 μM ARA. Under these conditions, a rapid multimerization was still observed shortly after the addition of ARA, as shown in Fig. 5a, and an increase in their numbers was present during the first 6 hours, resembling the timescales of the process at higher concentrations (Fig. 1a). The numbers of the detected oligomers, and their estimated concentrations (Supplementary Fig. 6b) were lower in comparison to the results in the high-concentration experiments, which highlights the challenge of monitoring this low-concentration process using more conventional bulk methods. Despite the lower overall numbers of oligomers, the growth of these species was again observed, with similar apparent size distributions compared to the higher-concentration reaction (Fig. 5b), and very similar FRET efficiency histograms (Fig. 5c). Further, we used CD to determine the conformation of these multimers. Preliminary attempts to measure the solutions containing 2 μM αS and 10 μM ARA resulted in the spectra indicating the presence of intrinsically disordered protein. This was consistent with the single-molecule observations that even though the multimers were present in the solutions, the majority of αS was still in its monomeric form, as is indicated by the low estimated concentrations of the multimers (Supplementary Fig. 6b). We therefore enriched the multimers using 100 kDa spin-filters, as described in Methods, and their CD spectrum indicated that these species were alpha-helically-folded, similarly to the species generated at 35 μM αS with 1 mM ARA (Fig. 5d). To note, the retention by the 100 kDa cutoff filter is consistent with the oligomers being the size of a tetramer and larger.

Based on these results, it could be concluded that the preparation at 2 μM αS and 10 μM ARA yielded the same species as the higher-concentration preparation, even though the overall number of multimers was low at these physiologically-relevant concentrations. To confirm this conclusion, we monitored the disaggregation of the low-concentration multimers, by diluting the samples to single-molecule concentrations and recording the decrease in their numbers over time due to their dissociation. The resulting disaggregation profiles were similar to the ones obtained for the high-concentration preparations at 35 μM αS and 1 mM ARA, as shown in Fig. 5e.

In order to test whether disease-associated point mutations of αS could lead to the self-assembly upon the action of ARA, we prepared the samples using 2 μM αS as well as 2 μM A30P, A53T and E46K mutants of αS, with the addition of 10 μM ARA. As a result, the formation of oligomers was observed for αS, as before. In the case of the mutants, however, the samples contained more aggregates than the wild-type (A90C) protein in the absence of ARA, and the presence of ARA had less effect on the number of aggregates, as shown in Fig. 5f. Thus, at the chosen conditions, the effect of ARA was comparable between wild-type αS and E46K mutant, and much less apparent for the pathological mutants A30P and A53T in comparison to the wild-type αS.

## Discussion

In this study, we have generated alpha-helical multimers of αS in the presence of ARA, and demonstrated their clear differences compared to the toxic beta-sheet-rich oligomers of αS, characterized in our previous works^35^,^38^. ARA-induced oligomers differed from the oligomers generated in the absence of ARA in both morphology and size, displayed lower stability towards the changes in buffer conditions and had a higher propensity to disassemble upon sample dilutions. In addition, they were more susceptible to enzymatic digestion and degradation by proteasome. Despite their apparent lower stability in comparison to the αS-only oligomers, we found that these multimers could preserve their conformation upon the reduction of ARA concentration, which is particularly significant considering that ARA is highly transient *in vivo*^44^.

**Figure 6:**
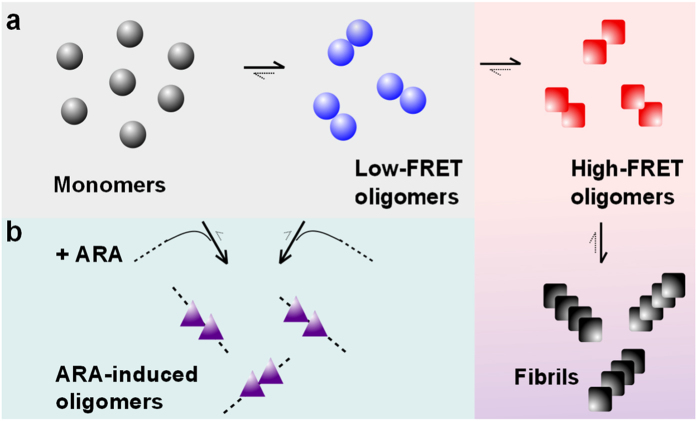
Schematic outline of the self-assembly mechanisms of αS in the absence and in the presence of ARA. (**a)** Without ARA, monomeric protein assembles according to the aberrant aggregation mechanism, as reported previously^38^. In this mechanism, αS forms disordered low-FRET oligomers, which convert to toxic and beta-sheet-rich high-FRET oligomers, which in turn convert into fibrils. (**b)** Upon the addition of ARA, the alpha-helical ARA-induced multimers are formed, comprising both αS and ARA. Equilibrium with monomer is consistent with the immediate ARA-induced oligomer formation, and equilibrium with low-FRET oligomers can account for the observed time-progression during first 6 hours of the reaction.

The ARA-induced oligomers in our experiments were evidently distinct from the beta-sheet-rich oligomers formed during the aberrant aggregation of αS. Moreover, they were resistant to fibril formation, as judged from TEM imaging, and required ARA for their stabilization, as concluded from the ARA washing experiments. These species may therefore represent the products of an alternative reaction involving both αS and ARA, as is schematically illustrated in Fig. 6. Because their formation is fast and recruits monomeric αS, it can compete with the slow formation of high-FRET beta-sheet oligomers particularly when the total monomer concentration is low. We previously showed that the beta-sheet-rich oligomers of αS were the most cytotoxic species^35^ and caused damage to neuronal cells when present even at picomolar concentrations^38^, and reconfirmed their toxicity in the present study. Therefore, any competing mechanisms that inhibit their formation may be highly neuroprotective. This is consistent with the finding that disease-associated mutants of αS had a lower tendency to assemble into the FA-induced species compared to the wildtype αS, which leaves them in a free state and may ultimately lead to the generation of higher concentrations of toxic beta-sheet-rich oligomers via the aberrant aggregation mechanism. To note, this *in vitro* result is in striking agreement with what was shown for PD mutants of αS in intact neurons by both cell-penetrant crosslinking and fluorescent protein complementation^48^.

Considering the remarkable ease of formation of the ARA-induced multimers and their stability under physiologically relevant concentrations, it can be argued that the formation of closely-related multimers may potentially occur *in vivo*. In line with this, multiple properties of the ARA-induced oligomers show close resemblance to the native alpha-helical multimers of αS extracted *ex vivo*^9^,^10^ such as being small, below 10-mers, alpha-helically-folded and aggregation-resistant. In our experiments, these multimers were present in the excess of monomeric αS, which was concluded from the observation of an excess of monomeric bursts in addition to the multimers during sm-FRET measurements (Supplementary Fig. 1), and from the CD spectra of the flow-through after their enrichment (Fig. 5d). This may suggest that these species are in equilibrium with monomeric αS, similarly to what was proposed for the native multimers^27^,^48^.

Clearly, although ARA has been used in the present study, numerous polyunsaturated FAs and their mixtures *in vivo* may play a similar role to facilitate the formation and stabilization of aggregation-resistant alpha-helical multimers of αS. This may contribute towards the reduction in cytotoxicity associated with these FAs^49^,^50^ in the context of PD and related disorders. Given the strong biophysical evidence for the formation of alpha-helical multimers of αS in the presence of ARA in our study, we therefore suggest that ARA and other polyunsaturated FAs may be the unidentified stabilizing co-factors for the native multimers of αS, suggested previously^51^. Thus, polyunstaturated FAs may play a crucial role in αS homeostasis via the stabilization of native multimers and the prevention of aberrant aggregation of αS.

## Methods

### Reagents

ARA, Trizma base, NaCl, CaCl_2_. Proteinase-K, THT and propidium iodide were purchased from Sigma Aldrich. Hoechst 33342 was from Molecular Probes. Alexa Fluor 488 C5 maleimide and Alexa Fluor 594 C5 maleimide dyes were from Life Technologies.

### ARA solution preparation

ARA was stored under nitrogen at –80 °C, and aqueous stock solutions were freshly prepared prior to all experiments by adding the pure FA to ice-cold buffer (25 mM Tris, 0.2 M NaCl) followed by vigorous agitation.

### α-Synuclein preparation

αS wild-type and A90C were expressed as previously described^52^. A90C was labeled with maleimide-linked Alexa Fluor 488 (AF488) or Alexa Fluor 594 (AF594) and separated from the unreacted dyes as previously reported^35^,^38^. The protein aliquots were stored at −80 °C and thawed once before use.

### α-Synuclein aggregation

For the preparations of αS oligomers in the absence of ARA, a 35 μM protein solution was made, containing 1:1 stoichiometric ratios of AF488- and AF594-labeled αS in Tris buffer (25 mM Tris, 0.1 M NaCl, pH 7.4), with 0.01% NaN_3_, and a total sample volume was 300 μL. The buffer in all single-molecule experiments was pre-filtered using 0.02 μm syringe filter (Anotop, Whatman). The aggregation mixture was incubated in the dark over 24 h at 37 °C with constant shaking at 200 r.p.m. (New Brunswick Scientific Innova 43, 25 mm orbital diameter), and subsequently centrifuged at 14.2 r.p.m. for 15 min and separated from fibrillar pellet.

For the preparations of ARA-induced oligomers of αS, an aggregation mixture contained 1:1 ratio of AF488- and AF594-labeled αS, with the total αS concentration of 35 μM, and 1 mM concentration of ARA, by diluting into Tris buffer (25 mM Tris, 0.1 M NaCl, pH 7.4) and 0.01% NaN_3_, with the total volume of 300 μL. The mixture was incubated in the dark over 24 hours at 37 °C without agitation.

### sm-FRET measurements and data analysis

Single-molecule confocal setup, the experimental procedure and data analysis were similar to previously reported^38^, and are detailed in Supplementary Information. FRET efficiency values were calculated according to


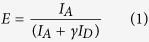


where *I*_*D*_ is the corrected donor fluorescence intensity, *I*_*A*_ is the corrected acceptor intensity and γ is a gamma factor (0.99) specific to the instrument. FRET efficiency values were represented as histograms with a bin-width of 0.05 (Fig. 3a). Oligomer apparent size was estimated using


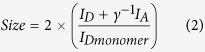


where *I*_*Dmonomer*_ was the average intensity of fluorescence bursts in the donor channel after exclusion of oligomeric bursts. In this equation, the total fluorescence intensity from AF488 (numerator) is normalized by the average AF488 monomer brightness. The factor of two corrects for the 1:1 stoichiomerty of AF488 and AF594 fluorophores. Species occupying multiple consecutive time-bins or determined to be greater than 150-mers were assumed to be either fibrillar or arising from dust, and removed from the analysis^36^. The expression is valid under the assumption that there is no appreciable quenching in the soluble oligomers, as was demonstrated in our previous work^35^. Note that the size distributions are referred to as “apparent”, serving as estimates owing to the stochastic nature of fluorescence emission and different paths the oligomers can take through the confocal volume.

In order to determine the average FRET efficiency value, the FRET histograms of selected timepoints were fitted to either a single or a double Gaussian distribution, depending on the presence of either one of two peaks, using GaussAmp functions:


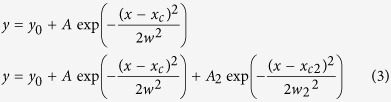


where *A* and *A*_*2*_ are the amplitudes, *x*_*c*_ and *x*_*c2*_ the centers, corresponding to average FRET efficiency values (E), *w* and *w*_*2*_ the widths of the distributions. The representative fitted histograms are shown in Fig. 3a.

### CD measurements

For experiments in Fig. 1c, samples were prepared in the same way as for the sm-FRET experiments, using AF594-labeled αS, and diluted into Tris buffer to the final protein concentrations of 3–10 μM prior to the measurements. CD spectra were recorded on Jasco J-810 spectropolarimeter, using a quarz cuvette of 1 mm. The spectra were acquired between 205 and 250 nm, with an interval of 0.2 nm, and an average of 10 accumulations per spectrum, using 1 nm bandwidth and a scanning speed of 50 nm/min. The spectra were corrected for the background from the buffer in the case of αS in buffer samples, and for the background from the buffer with ARA, for the samples containing ARA. FFT smoothing with a window of 25 datapoints was applied (Origin), and the resulting spectra were presented as millidergees versus wavelength. For experiments in Fig. 5d, samples containing 2 μM αS (1:1 AF488- αS and AF594- αS) and 10 μM ARA at a total volume of 300 μL were incubated for 24 h under the same conditions as before. Subsequently, 10 samples (3 mL) were combined and centrifuged in a filter device with a molecular cut-off of 100 kDa (Amicon Ultra, Millipore). It was expected that some of the ARA-induced oligomers would remain on top of the filter resulting in the multimer-enriched sample. The specta of both flow-through and the solution remaining on the filter were acquired using the same spectrometer and settings as before, between 200 and 250 nm and 20 accumulations per spectrum. The spectra were corrected for the background from buffer. No additional smoothing was applied, and the result was expressed as millidegrees versus wavelength. For the measurement in Fig. 4c (of the samples washed with buffer to remove excess ARA), the protein concentration was 4 μM, the spectrum was recorded using identical setting as above, with 20 accumulations per spectrum, and the spectrum of buffer was subtracted from the spectrum of the sample.

### TEM Imaging

For TEM imaging (Fig. 2), the sample preparation was the same as for sm-FRET experiments, and the samples were imaged after 24 h of incubation. 10 μL volumes of the samples were applied onto carbon-coated 400-mesh copper grids (Agar Scientific) for 5 min, and washed with double distilled water. Negative staining was carried out by using 2% (w/v) uranyl acetate. TEM images were acquired using Tecnai G2 microscope (13218, EDAX, AMETEK) operating at an excitation voltage of 200 kV.

### Oligomer stability at different ionic strengths

It was recently demonstrated using the sm-FRET method that αS oligomers had differential stabilities depending on the ionic strength of buffer solutions^36^. To compare the stability of the ARA-induced oligomers and αS oligomers formed in pure buffer with respect to the changes in ionic strength, the samples after 24 h of incubation were diluted 1:100,000 into either Tris, Tris/2 (Tris buffer diluted with MQ water), Tris/4, Tris/8 and Tris/16, and analyzed by sm-FRET immediately after dilution (Fig. 3c).

### Proteasome degradation assays

Mammalian 26S proteasomes were purified from HEK293T cells overexpressing rpn11-His-TEV-biotin acceptor sequence (kind gift from Lan Huang, UC Irvine) and purified using established protocols^53^,^54^. Cells were grown until 100% confluent and collected and resuspended with a scraper in ice-cold Proteasome buffer (50 mM Tris, pH 7.5, 0.5% NP-40, 10% glycerol, 5 mM ATP, 1 mM DTT, 5 mM MgCl_2_). Dounce homogenizer was then used for cell lysis and the lysate was cleared by centrifugation at 3000 × g for 5 min at 4 °C. This lysate was subsequently incubated with 2 ml bed volume of NeutrAvidin beads (Pierce) at 4 °C overnight. Unbound proteins were washed off with 20 ml proteasome buffer and bound proteasomes were cleaved off the column with 6 μl of TEV protease (Invitrogen). Proteasomes were concentrated to >2 μM and frozen in aliquots for single use. For the comparison between the ARA-induced oligomers and αS oligomers formed in pure Tris buffer with respect to their stability towards degradation by 26S proteasome, samples containing 35 μM αS in buffer, or 35 μM αS plus 1 mM ARA after 24 h of incubation were diluted 1:3.75 for proteasomal degradation. The final assay contained 40 nM proteasome, 125 mM ATP.MgCl_2_, 5 μM creatine kinase and 0.1 M creatine phosphatase in 50 mM Tris buffer (pH 7.4). The resulting samples were analyzed by sm-FRET both immediately after mixing, and after incubation for 12 h under quiescent conditions at 37 °C. The fractions of non-degraded oligomers were determined as the numbers of oligomers after the incubation divided by the numbers of oligomers immediately after mixing (Fig. 3d).

### Oligomer disaggregation upon dilution

To further compare the stabilities of ARA-induced oligomers and oligomers of αS formed in pure buffer, both types of samples after 24 h of incubation were diluted 1:100,000 into Tris buffer of the same composition as for the aggregations, incubated at quiescent conditions at ambient temperature in low-binding test-tubes (Protein LoBind, Eppendorf), and regular aliquots were analyzed by sm-FRET over 7 h after dilution, ensuring that the aliquots were withdrawn for the analysis at the same incubation time for either type of samples, to allow a comparison of the disaggregation reactions (Fig. 3e). The same experiment was carried out using ARA-induced oligomers prepared at 2 μM αS and 10 μM ARA, and compared with the disaggregation profiles of the ARA-induced oligomers generated at high concentration, as show in Fig. 4e.

### Proteinase-K digestion assays

To carry out further structural comparison between the ARA-induced oligomers and αS oligomers formed in pure Tris buffer, their susceptibility to Proteinase-K (PK) digestion was investigated (Fig. 3f). Beta-sheet structure, present in fibrils and high-FRET oligomers, is resistant to PK digestion, as was shown in our previous works^35^. PK aliquots were prepared in Tris buffer defined above, with the addition of 1 mM CaCl_2_, and stored at −80 °C before use. Samples after 24 h incubation were diluted into a range of PK concentrations between 0–10 μg/ml in Tris buffer with 1 mM CaCl_2_. incubated at 37 °C for 5 min, and subsequently further diluted for the sm-FRET analysis.

### Depletion of ARA concentration

ARA-induced oligomers were prepared as described above, and subsequently the concentration of free ARA was decreased by washing with copious amounts of buffer, and the protein was concentrated using a spin filter with a molecular cutoff of 5 kDa (Sartorius). Based on the total volume of buffer used for washing in this experiment, the concentration of ARA would be reduced to less than 500 nM. Note that this estimation is a lower bound and does not account for the ARA binding to αS. This preparation is referred to as “ARA washed” in Fig. 4.

### Cell culture

Mixed cultures of cortical neurons and glial cells were prepared and cultured as described previously^55^. The BV2 cell lines were derived from immortalized murine neonatal microglia. They were grown and incubated at 37 °C in a humidified atmosphere of 5% CO_2_ and 95% air, until ~300,000 cell/ml.

### Protein sample preparation for the cell assays

For the assays, unlabeled wild-type αS was used instead of dual-labeled samples in order to ensure the absence of fluorescence emission from AF labels. Protein sample preparation protocol was identical to the procedures for sm-FRET experiments. The solutions after 24-h incubation were applied on cells at the same total concentration of αS, and at the corresponding concentration of ARA.

### ROS measurements

The experiments (Fig. 4d) were carried out according to previously detailed protocols^35^ as detailed in Supplementary Information, and involved measurements within first 10 minutes upon the sample application to the cell cultures.

### Cell death assays

In these experiments (Fig. 4e) αS and ARA solutions were applied to primary co-cultures overnight at 37 °C. The cells were loaded simultaneously with 20 μM propidium iodide, which is excluded from viable cells but exhibits red fluorescence following a loss of membrane integrity, and 10 μM Hoechst 33342 (Molecular Probes), which gives blue staining to chromatin, and the total number of dead cells was counted, as further detailed in Supplementary Information and Supplementary Fig. 5.

### Quantification of TNF-α production

The experiments in Fig. 4f were carried out using BV2 microglia. αS-only and ARA-induced oligomer solutions as well as free ARA were prepared and applied at a range of concentrations from 0.05–200 μM of αS. After application, the cells were incubated at 37 °C for 24 h, and the supernatants were subsequently collected and analyzed using TNF-α Elisa kit (R & D, Minneapolis, MN) according to manufacturer’s protocol. In this assay, an increase in TNF-α due to free ARA (59.673 pg/mL) could be detected only upon application of excess ARA (1 mM) and 5-d incubation.

### Statistical analysis of the data from cell experiments

Student t-tests were carried out using Origin 9 software (Microcal Software) and the resulting p values are reported in the legend of Fig. 4.

### Comparison of ARA-induced multimerisation using pathological mutants of αS

In order to investigate whether the formation of ARA-induced oligomers could be observed at low concentrations when using pathological mutants of αS, dual-labelled A90C αS was used, and dual-labelled A30PA90C, A53TA90C and E46KA90C αS variants, denoted as A30P*, A53T* and E46K* in Fig. 4f, where ‘*’ indicates that these isoforms carry the A90C mutation for the fluorophore incorporation in addition to the disease-associated mutation. The pathological mutants were expressed and purified as previously described in detail. Samples of 2 μM αS of every isoform in the presence of 10 μM ARA, or in its absence (6 separate samples in each case) were prepared and incubated at 37 °C without shaking for >30 h to allow steady-state aggregate populations, and subsequently analysed by sm-FRET, keeping the protein concentration for the detection the same for all samples, as judged by comparing the monomer burst rates. Numbers of detected aggregates were compared (Fig. 5f).

## Additional Information

**How to cite this article**: Iljina, M. *et al*. Arachidonic acid mediates the formation of abundant alpha-helical multimers of alpha-synuclein. *Sci. Rep*. **6**, 33928; doi: 10.1038/srep33928 (2016).

## Supplementary Material

Supplementary Information
