# Supplementary material for: Arachidonic acid mediates the formation of abundant alpha-helical multimers of alpha-synuclein
Source: Sci Rep. 2016 Sep 27;6:33928. doi: 10.1038/srep33928 (PMC5037366; doi:10.1038/srep33928)
Supplement: Supplementary Information [file srep33928-s1.pdf]

Supplementary Information

Arachidonic acid mediates the formation of abundant alpha-helical multimers of alpha-synuclein

Marija Iljina, Laura Tosatto, Minee L. Choi, Jason C. Sang, Yu Ye, Craig D. Hughes, Clare E. Bryant, Sonia Gandhi and David Klenerman*

* Correspondence may be addressed to: dk10012@cam.ac.uk

Supplementary Methods

sm-FRET measurements and data analysis. Aliquots from the aggregation samples were diluted to single-molecule concentrations prior to the measurement in Tris buffer of the same composition as what was used for the sample preparations. The diluted αS sample was introduced into a straight channel of a microfluidic device (PDMS, 25 µm height, 100 µm length), using a 200 µL gel-loading tip as a vessel. A collimated 488 nm laser beam (Spectra Physics NewPort Cyan) was directed through a back port of an inverted microscope (Nikon Eclipse Ti-U) at 2 mW power (measured at the back port of the microscope), where it was reflected by a dichroic mirror (Semrock DiO1 R405/488/594) and sent through an oil immersion objective (Plan Apo VC 60 x, NA 1.40, Nikon) to be focused 10 µm into the center of the microfluidic channel. Fluorescence signal was collected by the same objective, imaged onto a 100 µm pinhole (Thorlabs) and separated into two channels by a dichroic mirror (Horiba 585DRLP). Donor fluorescence was filtered by a long-pass (Edge Basic 514) and a band-pass filter (535AF45 Omega Filters) before being focused onto an avalanche photodiode, APD (Perkin Elmer). Acceptor fluorescence was directed through a long-pass filter (610ALP Horiba) and a band-pass filter (BrightLine 629/53) before being focused onto a second APD. Synchronous output from the APDs was collected by custom-implemented field-programmable gate array, FPGA (Celoxica RC10). The data consisted of time-binned photon bursts in the donor and the acceptor channel (schematically illustrated in Supplementary Figure 1), and were collected with a 50 µs bin-width, 100,000 bins per frame, 40 frames, and a total duration of 200 s, or for longer (up to 900 s) in the cases where the samples contained low amounts of oligomeric species. To note, the final concentration for the detection and the total duration of data collection were always kept the same in the cases where comparison between different samples was carried out. Igor Pro (Wavemetrics) custom-written code was used to analyze the data, as previously described[1]. Time-bins with intensities greater than 15 photons/bin in the donor and the acceptor channel simultaneously (the AND criterion)[2] were selected and assigned to the events arising from oligomers. The number of these events was counted within the chosen detection for an aliquot, and assigned as the number of oligomers per sample. The intensities of the selected oligomeric events were corrected for the AF488-AF594 cross-talk and background fluorescence, and used to calculate FRET efficiency values and apparent sizes, using equations 1 and 2 in Methods (main text). It was additionally verified that ARA blank solutions were fluorescently inactive both shortly after sample preparation and following incubation.

Determination of the optimum conditions for the preparation of ARA-induced oligomers and oligomers in buffer. Based on the results of sm-FRET experiments (Fig. 1 in the main text), ARA was efficient at mediating the formation of αS aggregates at quiescent conditions at 1 mM concentration. The preparations of the ARA-induced oligomers and the oligomers formed in its absence have been optimized in order to carry out the sm-FRET comparison of these species, as described. Rapid ARA-induced oligomer formation was observed in these experiments when using an A122C mutant of αS instead of the A90C, where fluorophore is attached to a molecule of αS at residue 122 instead of residue 90 (Supplementary Fig. 3).This indicates that this effect is not specific to the chosen A90C isoform of αS, and also may suggest that neither residue 122 nor 90 are directly involved in the binding to the FA. It is interesting to note that previously, the residues 2-19 and 123-140 in the αS molecule were identified to be homologous with a motif of fatty acid binding proteins[3]. The absence of any inhibition of the ARA-promoted oligomerization in the case of incorporating the fluorescent dye at the residue 122, neighboring with 123, may suggest that the ARA binding region in the C-terminal of αS is either absent or comprises later residues. Since there was no difference in the acid-induced oligomer formation under either quiescent or shaking conditions, all subsequent ARA-induced oligomer preparations were carried out without shaking. In the case of control

samples, 35 µM αS in Tris buffer, the oligomerization was more efficient under shaking conditions, reflected by the higher numbers of the detected species under these conditions (Supplementary Fig. 3), and therefore the subsequent preparations of αS oligomers in buffer were carried out with shaking. Typically, the observed numbers of oligomers were much higher in the samples containing ARA compared to the samples without the acid, indicating that the oligomers promoted by ARA were much more abundant. In addition, the aggregations using 35 µM αS and 1 mM ARA yielded the highest concentrations of ARA-induced oligomeric species amongst several other attempted combinations, summarized in Supplementary Figure 4.

Control experiments to investigate the photophysical effects of ARA on AF dyes. DNA duplexes dual-labeled with Alexa Fluor (AF) dyes were used. For the preparation of the DNA duplexes, synthetic complementary pairs of oligonucleotides were purchased from ATDBio Ltd. (Southampton, UK), and were purified by the supplier by double HPLC. The sequences of the oligonucleotides were:

TCCD olig 1: Alexa Fluor 488-TAGTGTAACTTAAGCCTAGGATAAGAGCCAGTAATCGGTA

TCCD olig 2: Alexa Fluor 594-TACCGATTACTGGCTCTTATCCTAGGCTTAAGTTACACTA

FRETolig 1: TACTGCCTTTCTGTATCGC-Alexa Fluor 488-
-TATCGCGTAGTTACCTGCCTTGCATAGCCACTCATAGCCT

FRETolig 2: AGGCTATGAGTGGCTATGCAAGGCAGGTAACTACGCGATAAGCGATACAGA-Alexa Fluor 594

The dual-labeled duplexes were formed by heating a 1:1 mixture of either TCCD olig 1 and 2, or FRET olig 1 and 2 at a total concentration of 1 µM to 95 °C, and slowly cooling to room temperature. The duplexes were diluted to single-molecule concentrations into Tris buffer of the same composition as used for the aggregation preparations, either in the presence or in the absence of ARA. Solutions containing TCCD DNA duplex were measured using a dual laser excitation, by illuminating the samples with overlapped 488 nm and 594 nm laser beams. FRET DNA duplex had the dyes close enough for the FRET process to occur; therefore the experiments were carried out using the 488 nm laser excitation, according to the same protocol as what was used for the sm-FRET measurements of the dual-labeled protein samples. In both cases, the numbers of the recorded coincident events in the donor and the acceptor channels were counted for the samples in pure buffer, and in the presence of ARA, and were compared (Supplementary Figs. S2a and b). In addition, the average intensity of the fluorescence bursts in both the donor and the acceptor channels was compared (Supplementary Fig. 2c).

ROS measurements. The experiments were carried out according to previously detailed protocols[4,5]. For the assays, unlabeled wild type αS was used, and ARA-induced oligomers and αS oligomers only were prepared and incubated under identical conditions as for sm-FRET experiments. After 24-h incubation, the solutions were withdrawn and used for reactive oxygen species (ROS) experiments. The solutions were applied to primary co-cultures of neurons and astrocytes loaded with dihydroethidium (HEt) (2 µM HEt was present in the solution during the experiments). No preincubation ("loading") with HEt was used in order to limit the intracellular accumulation of oxidized products. For HEt measurements, a ratio of the fluorescence intensities resulting from its oxidized/reduced forms was quantified. Emission recorded above 560 nm was assigned to be from the oxidized form (ethidium), while emission collected from 405 to 470 nm was from the reduced form (hydroethidium). The rate of ROS production was determined by dividing the gradient of the HEt ratio function against time after application of 500 nM αS solution by the gradient prior to αS application. The results are summarized in Fig. 4d, main text.

Cell death assays. These assays were carried out to investigate whether the ARA-induced oligomers had the ability to induce cell death. We applied the solutions prepared as before, and incubated the treated cultures overnight. The cells were loaded simultaneously with 20 μM propidium iodide (PI), which is excluded from viable cells but exhibits red fluorescence following a loss of membrane integrity, and 10 μM Hoechst 33342 (Molecular Probes), which gives blue staining to chromatin, to count the total number of cells. The results of these assays are in Fig. 4e, main text. The incubation of cells with 500-nM αS-only samples resulted in a significant increase in cell death, while the incubation of cells with ARA-induced oligomers after ARA depletion did not alter the basal rate of cell death.

Supplementary Results

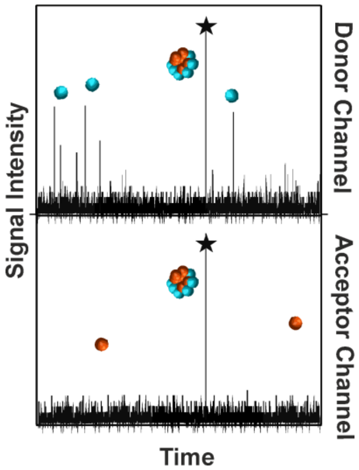

Supplementary Figure 1. Illustrative sm-FRET fluorescence intensity traces, when 488-nm excitation is used. Non-coincident bursts in the donor channel arise from the AF488-labeled αS monomers (represented as blue spheres), and are typically present in excess, while the AF594-labeled monomers (orange spheres) are not detected. The simultaneous intensity burst, marked with black stars, corresponds to the αS oligomer.

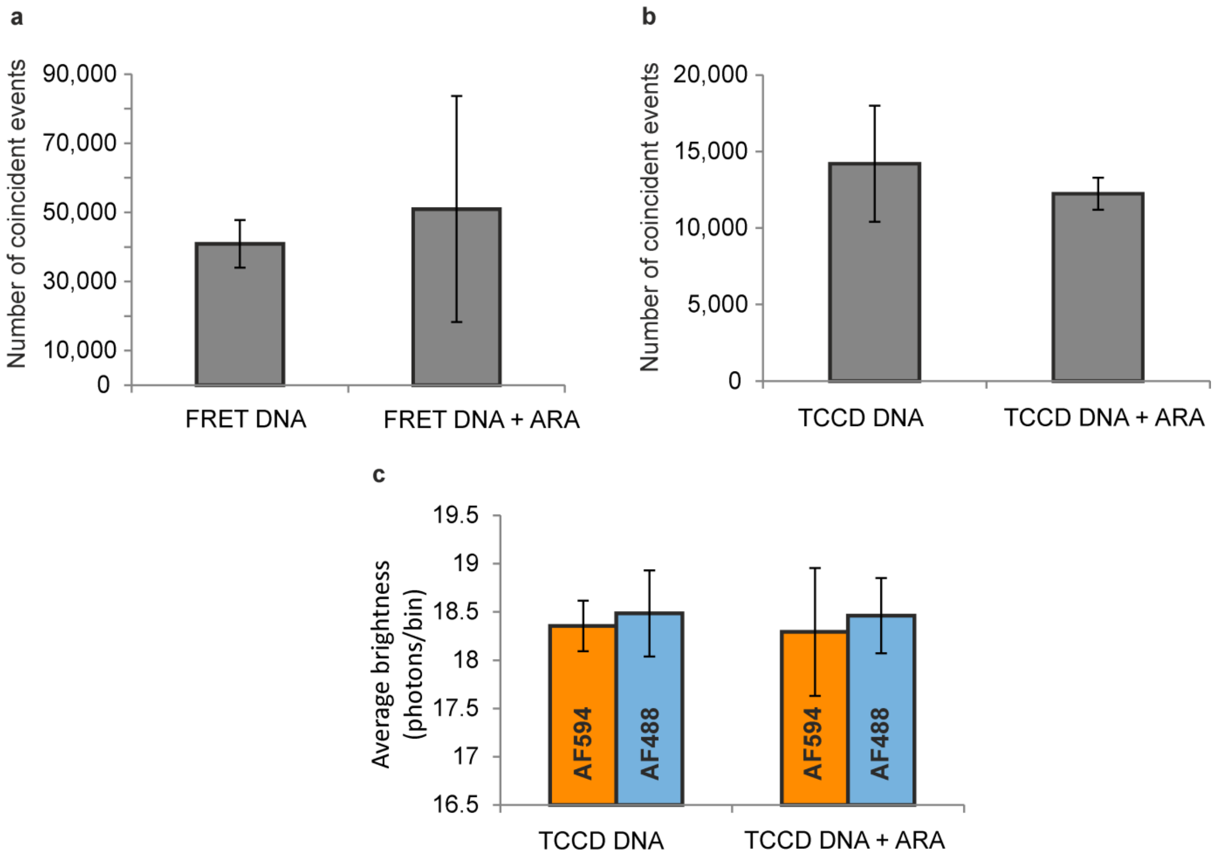

Supplementary Figure 2. Control experiments to investigate the photophysical effects of ARA on AF dyes in the single-molecule experiments. **a.** and **b.** Number of coincident events recorded for either FRET or TCCD DNA duplexes diluted into pure buffer, or in the presence of ARA (n=5, std).
c. Average intensity of the fluorescence bursts in both the donor and the acceptor channels, derived from TCCD experiments with dual-color excitation.

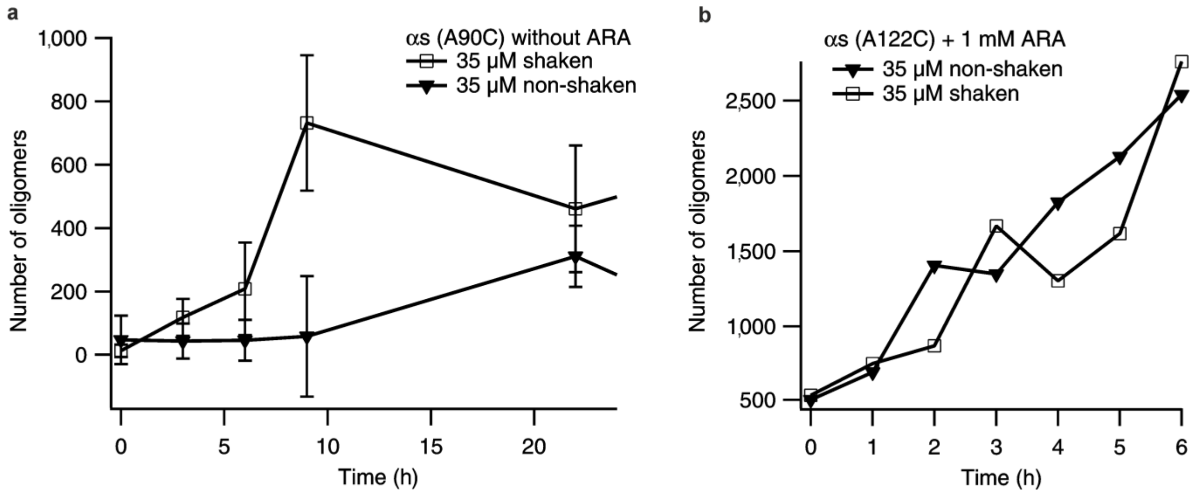

Supplementary Figure 3. Control aggregation experiments. a. 35 μM αS in buffer. Aggregation is promoted by shaking, therefore shaking conditions are chosen for αS–only oligomer production in all further experiments (n=3, std). **b.** 35 μM A122C isoform of αS in the presence of 1 mM ARA(n=3). Comparable results under either shaking or non-shaking conditions, similarly to the results with A90C mutants of αS, shown in Fig. 1d (main text).

a

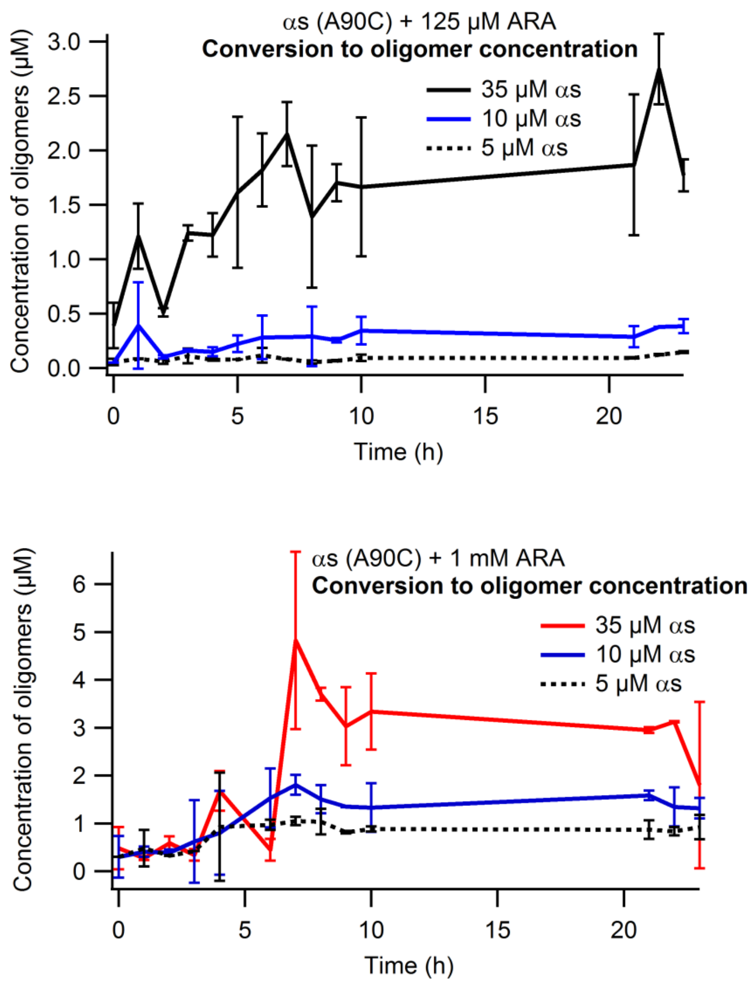

b

Supplementary Figure 4. Kinetic profiles to determine the high-yielding conditions for ARA-induced oligomer production (n=3, std in each case). In **a.** and **b.**, oligomer concentrations were approximately estimated in each case, by normalizing to the monomeric bursts (twice the bursts in the donor channel) to estimate the fraction of oligomers, and subsequent multiplication by the bulk starting total protein concentration. The highest estimated concentration of oligomers was generated at 35 µM αS in the presence of 1 mM ARA.

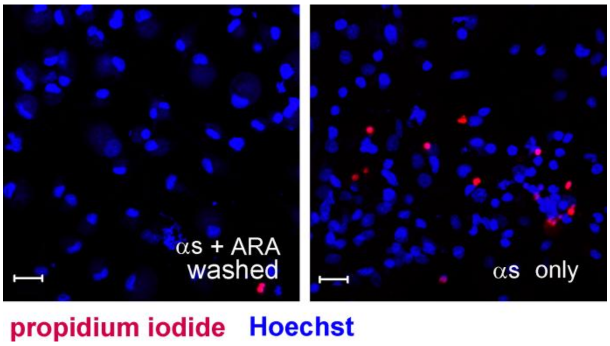

Supplementary Figure 5. Representative images from cell death assays in main text, Fig. 4e. Overnight incubation with 500 nM of αS-only oligomer solution resulted in increased cell death, while 500 nM sample of ARA-induced oligomers of αS resulted only in basal levels of cell death. In this experiment, dead cells are stained in pink with propidium iodide, enabling their quantification. Scale bar is 20 µm.

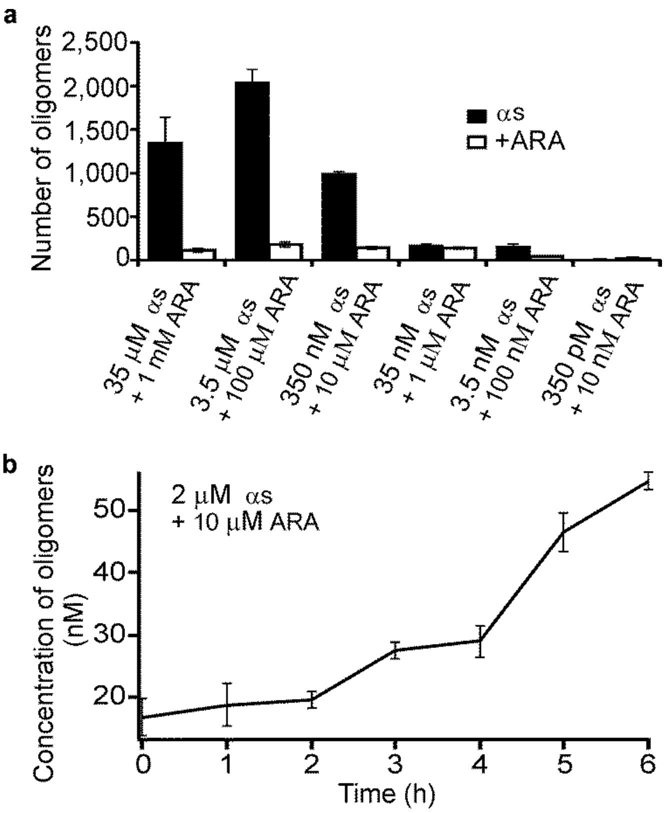

Supplementary Figure 6. Extension towards lower concentrations of αS and ARA. The same sample preparation procedure was used as for the preparation of ARA-induced oligomers in the previous experiments, but the total concentrations of both αS and ARA were gradually decreased. **a.** Keeping the same ratio of ARA:αS, the induction of oligomerisation is observed reproducibly starting from 10 µM of ARA (n=3, std). As control samples, αS in buffer solutions were incubated in the absence of ARA under the same conditions (n=3, std). **b.** Kinetic profile of oligomer formation at 2 µM αS with 10 µM ARA, converted to approximate oligomer concentrations as in Supplementary Figure 4.

Supplementary References

1 Horrocks, M. H. *et al.* Fast Flow Microfluidics and Single-Molecule Fluorescence for the Rapid Characterization of α-Synuclein Oligomers. *Anal Chem* **87**, 8818-8826, doi:10.1021/acs.analchem.5b01811 (2015).

2 Ying, L., Wallace, M., Balasubramanian, S. & Klenerman, D. Ratiometric analysis of single-molecule fluorescence resonance energy transfer using logical combinations of threshold criteria: A study of 12-mer DNA. *Journal of Physical Chemistry B* **104**, 5171-5178, doi:10.1021/jp993914k (2000).

3 Sharon, R., Goldberg, M. S., Bar-Joseph, I., Shen, J. & Selkoe, D. J. alpha-Synuclein occurs in lipid-rich high molecular weight complexes, binds fatty acids and shows homology to the fatty acid binding proteins. *Society for Neuroscience Abstracts* **27**, 608 (2001).

4 Cremades, N. *et al.* Direct observation of the interconversion of normal and toxic forms of α-synuclein. *Cell* **149**, 1048-1059, doi:10.1016/j.cell.2012.03.037 (2012).

5 Deas, E. *et al.* Alpha-Synuclein Oligomers Interact with Metal Ions to Induce Oxidative Stress and Neuronal Death in Parkinson's Disease. *Antioxid Redox Signal* **24**, 376-391, doi:10.1089/ars.2015.6343 (2016).
